# Reduced-intensity conditioning allogeneic stem cell transplantation in malignant lymphoma: current status

**DOI:** 10.7497/j.issn.2095-3941.2013.01.001

**Published:** 2013-03

**Authors:** Le Zhang, Yi-Zhuo Zhang

**Affiliations:** Department of Hematology, Tianjin Medical University Cancer Institute and Hospital, Key Laboratory of Cancer Prevention and Therapy, Ministry of Education; Key Laboratory of Cancer Prevention and Therapy, Tianjin; State Key Laboratory of Breast Cancer Research, Tianjin 300070, China

**Keywords:** Reduced intensity conditioning (RIC), allogeneic stem cell transplantation (RIC-allo-SCT), Hodgkin’s lymphoma, indolent lymphoma, aggressive lymphoma

## Abstract

Allogeneic stem cell transplantation (allo-SCT) is a potential cure for patients with malignant lymphoma that is based on the graft-versus-lymphoma (GVL) effect. Myeloablative conditioning allo-SCT is associated with high mortality and morbidity, particularly in patients older than 45 years, heavily pretreated patients (prior hematopoietic stem cell transplantation or more than two lines of conventional chemotherapy) or patients affected by other comorbidities. Therefore, conventional allo-SCT is restricted to younger patients (<50 to 55 years) in good physical condition. Over the last decade, allo-SCT with reduced-intensity conditioning (RIC-allo-SCT) has been increasingly used to treat patients with lymphoma. This treatment is associated with lower toxicity and substantial decrease in the incidence of transplant-related mortality, and has the potential to lead to long-term remissions. Therefore, patients who are not suitable to undergo conventional allo-SCT can benefit from the potentially curative GVL effects of allo-SCT. Although RIC-allo-SCT has improved the survival of lymphoma patients, high post-transplant relapse rates or disease progression mainly results in treatment failure. Thus, further improvement is clearly needed. The role and timing of RIC-allo-SCT in the treatment of lymphoma remains unclear. Therefore, more prospective studies should clarify the effectiveness of this method. In this article, we review the recent literature on RIC-allo-SCT as a treatment for major lymphoma subtypes. Areas that require further investigation in the context of clinical trials are also highlighted.

## Introduction

The introduction of allogeneic transplantation (allo-SCT) has led to a remarkable progress in the treatment of lymphoma. Although outcomes have improved, standard myeloablative conditioning (MAC) allo-SCT is still associated with high transplant-related mortality (TRM) (20% to 60%) based on the literature^1^^-^^3^. The decreased risk of relapse compared with that in autologous transplantation is offset by a high TRM. Thus, MAC-allo-SCT has no clear advantage over autologous transplantation, and the high TRM rates restrict the use of this method to a minority of young and fit patients^4^. However, a significant shift in the process of allo-SCT has occurred over the last decade. The increasing number of patients receiving less toxic, reduced-intensity conditioning (RIC) regimens has broadened the applicability of this therapeutic approach. Allo-SCT with RIC greatly reduces TRM and improves overall survival (OS) without a commensurate increase in the risk for relapse^4^, resulting in the constant increase in the number of patients receiving RIC-allo-SCT^5^. Older patients or patients who underwent organ dysfunction pre-transplant and are unsuitable for high-dose chemotherapy should receive RIC-allo-SCT considering the transplant-related risk of allo-SCT^5^. We present in this article the analysis of data and the updated results for RIC-allo-SCT in different lymphoma subtypes.

## RIC-allo-SCT in Hodgkin’s lymphoma (HL)

The majority of patients with HL are cured using conventional chemoradiotherapy. High-dose therapy (HDT) followed by autologous stem cell transplantation (ASCT) is the standard of care for medically fit patients with relapsed HL^6^. The results of HDT/ASCT depend on different prognostic factors. The most important prognostic factors are clinical stage at relapse, tumor sensitivity to salvage chemotherapy, and remission duration between first-line treatment and relapse. The outcome is generally poor for patients relapsing after ASCT. If the disease progresses during HDT, relapsed patients who cannot benefit from ASCT should be considered for allo-SCT.

Early registry data^7^ show that allo-SCT after MAC results in lower relapse rates (RRs) but with significantly higher toxicity than ASCT. Studies from the 1990s suggest that the application of allogeneic strategies in patients with relapsed/refractory HL is limited by high nonrelapse mortality (NRM) varying from 40% to 60%^7^. The high procedure-related morbidity and mortality also prevents the widespread use of allo-SCT. The introduction of RIC regimens addresses this problem by reducing NRM while providing the GVL effect. The introduction of allo-SCT after RIC for relapsed/refractory HL patients results in a decreased cumulative incidence of NRM ranging from 11% to 13%. However, approximately 50% of all patients who undergo RIC-allo-SCT relapse^8^.

The Lymphoma Working Party (LWP) of the European Group for Blood and Marrow Transplantation (EBMT), together with the Grupo Español de Linfomas/ Trasplante Autólogo de Médula Ósea, conducted the largest multicenter phase II prospective clinical trial that aimed to analyze NRM and other major outcome parameters after RIC-allo-SCT in relapsed HL^9^. This study included 92 patients with relapsed HL, among which 14 died from progressive lymphoma before transplantation, and 78 continued with allograft (unrelated donors, *n*=23). The RIC regimens consisted of fludarabine (150 mg/m^2^ IV from day -8 to day -4) and melphalan (140 mg/m^2^ IV from day -3 to day -2). In addition, anti-thymocyte globulin (ATG) (45 mg/kg IV from day -4 to day -2) was used as graft-versus-host-disease (GVHD) prophylaxis in the recipients of stem cells obtained from matched unrelated donors (MUD). The NRM at 100 days and 1 year was 8% and 15%, respectively. For the allografted population, the 1-year and 4-year progression-free survival (PFS) rate was 48% and 24%, respectively. Patients allografted in complete remission (CR) exhibited remarkably better results, and 1-year and 4-year OS was 71% and 43%, respectively. Chronic GVHD (cGVHD) was associated with lower RRs (*P*=0.04). A significant improvement in PFS was also found in patients developing cGvHD (*P*=0.05). Donor type and stem cell source had no significant influence on post-transplant outcomes in HL patients, and the results were similar for sibling and MUD transplants. Although the RIC regimens significantly improved the survival outcomes in HL patients compared with the myeloablative strategies, the high relapse or progression rates are major challenges. Robinson *et al.*^8^ retrospectively investigated 285 adult relapsed/refractory HL patients undergoing different RIC regimens. They found that 89% of the patients were <45 years old and had chemosensitive disease. Furthermore, 80% of the patients had previously undergone ASCT, and 25% had refractory disease at transplant. Fludarabine/melphalan (FluMel), busulfan/fludarabine (BuFlu), and fludarabine/cyclophosphamide (FluCy) alone or with thiotepa were used as conditioning regimen. The early and cumulative 3-year TRM rates were 11% and 21%, respectively, and the 5-year progression rates reached up to 59%. The 3-year OS and PFS rates were 29% and 25%, respectively^10^. Researchers found that RR was lower for patients with GVHD. Relapse within 6 months of prior ASCT was associated with higher RR and lower PFS. Comparable results were observed in previous studies on the use of RIC regimens in HL patients. Despite the lower TRM rates, the OS and disease-free survival were not more than 50%^4^^,^^7^. Current data suggest that the chemosensitivity and CR status at transplant are the most important factors in improving survival after allo-SCT. Moreover, RIC-allo-SCT may be an effective salvage strategy for HL patients. However, the main cause of treatment failure following RIC-allo-SCT is disease progression.

## RIC allo-SCT in non-Hodgkin’s lymphoma

### Chronic lymphocytic leukemia (CLL)

Despite the often slow progression and good responsiveness of CLL to cytoreductive treatment and early intensive immunochemotherapy, approximately 20% of “poor-risk” patients^11^ show an aggressive course and die within a few years^10^. Based on the European EBMT CLL Transplant Consensus^12^, the criteria for poor-risk disease are non-response or early relapse within 12 months after purine analogue-containing therapy, relapse within 24 months after purine analogue combination therapy or treatment with similar efficacy (i.e., ASCT), and p53 deletion/mutation (del 17p-) requiring treatment. In 2006, the EBMT stated that allo-SCT is a reasonable treatment option for eligible patients with previously treated, poor-risk CLL. Allo-SCT can provide long-term disease control and has a potential curative role in patients with poor-risk CLL^13^.

Previous registry analyses of MAC-allo-SCT for CLL show that TRM rates range from 38% to 50%, and NRM rates can reach up to 44%. More recently published data indicate that the NRM of RIC regimens are between 15% and 25%^14^^-^^19^. The introduction of RIC-allo-SCT in the 1990s is a major progress in reducing the morbidity and mortality of allo-SCT. In Europe, the total number of allogeneic transplantations for CLL has decreased in the last seven years. In the last decade, many reports that primarily enrolled patients with chemorefractory end-stage disease have stressed the potential curative role of RIC-allo-SCT in CLL.

EBMT conducted a retrospective analysis on allo-SCT in 44 patients with del 17p- CLL. The majority (89%) of the patients in this study had received RIC. The results showed a 3-year PFS of 37% [95% confidence interval (CI), 22% to 52%], with no event occurring later than 3.5 years after transplantation (median follow-up, 39 months)^20^. Patients with bulky lymphadenopathy (>5 cm) at transplantation had significantly poor outcome, with a 5-year RR and PFS of 71% and 8%^18^. Dreger *et al.*^19^ performed a prospective clinical trial on RIC-allo-SCT in 90 patients with CLL. The conditioning regimen included fludarabine, and cyclophosphamide plus ATG in alternative donor transplants (*n*=65); alemtuzumab, fludarabine, and 2 Gy total body irradiation (TBI) (*n*=12); or fludarabine, busulfan, and cyclophosphamide plus ATG in alternative donor transplants (*n*=12). The 4-year PFS, OS, and NRM were 42%, 70%, and 21%, respectively. The results coincided with other prospective RIC studies^14^^-^^18^. These larger RIC prospective trials provide relatively concordant results. RIC-allo-SCT can highly improve the prognosis of poor-risk CLL patients. Nevertheless, the outcomes are considerably impaired if the disease is not in remission at the time of transplantation because of nodal bulks or chemotherapy resistance. Delgado *et al.*^21^ reported that using rituximab in addition to RIC-allo-SCT may facilitate disease control because of the direct cytotoxicity and modulation of the GVL effect. However, unresponsive, active, and bulky diseases at the time of transplantation result in poor results^15^^,^^19^, and disease relapse is still the primary cause of failure after RIC-allo-SCT in CLL patients. Therefore, the optimum choice of conditioning regimens should be based on the patient’s individual situation. MAC regimens may be preferable in younger patients with good performance status but poorly controlled disease, whereas RIC regimens are more appropriate in the presence of comorbidity and sensitive diseases^12^.

The German CLL Study Group is currently performing a trial to confirm the EBMT criteria in patients with high-risk and very high-risk CLL. Patients with high-risk disease (second EBMT criterion) who have a human leukocyte antigen (HLA)-identical donor are statistically randomized to allo-SCT while undergoing conventional salvage therapy. Patients with very high-risk disease (first and third EBMT criteria) undergo biological randomization based on donor availability. This trial may provide suggestions on when and how to use allo-SCT in the treatment of poor-risk CLL^13^ (**Table 1**).

**Table 1:** Results of prospective clinical trials on RIC-allo-SCT in CLL

Author	*n*	Conditioning regimen	4-y PFS	4-y OS	4-y NRM	Extensive cGVHD	Median follow-up y (range)
Sorror *et al.*^18^	82	F/TBI2^b^	39% (5y)	50% (5y)	23% (5y)	49%-53%	5
Brown *et al.*^15^	46	FB^c^	34% (2y)	54% (2y)	17% (2y)	38%	1.7
Khouri *et al.*^17^	39	FCR+/- ATG^d^	44%	48%	n.r.	58%	2.3 (0.3-6.7)
Schetelig *et al.*^14^	30	FB/ATG^e^	58%	69%	15%	21%	3.7 (2.1-5.6)
Delgao *et al.*^16^	41	FM/CD52^f^	45% (2y)	51% (2y)	26% (2y)	5% (2y)	1.3 (0-5.2)

PFS, progression-free survival; OS, overall survival; NRM: non-relapse mortality; y, year. ^a^Fludarabine and cyclophosphamide plus anti-thymocyte globulin (ATG) in alternative donor transplants (*n*=65); alemtuzumab, fludarabine, and 2 Gy total body irradiation (*n*=12); or fludarabine, busulfan, and cyclophosphamide plus ATG in alternative donor transplants (*n*=12); ^b^Fludarabine and 2 Gy total body irradiation; ^c^Fludarabine and busulfan; ^d^Fludarabine, cyclophosphamide, and rituximab plus ATG in alternative donor transplants; ^e^Fludarabine, busulfan, and ATG; ^f^Fludarabine, melphalan, and alemtuzumab.

### Indolent B-cell lymphoma

The median survival time for indolent lymphoma is 8 to 10 years. Although clinical advances are slow, several therapies are available. However, this disease is difficult to cure using conventional chemotherapy. Allo-SCT may be the only effective treatment for patients with this disease^5^. Based on epidemiological data, follicular lymphoma (FL) comprises more than 80% of the indolent lymphomas, and rituximab combined with various chemotherapy programs has been recommended for first-line therapy^22^. In addition, Khouri *et al.*^23^ reported that rituximab exhibits some activities in the treatment of GVHD and is increasingly incorporated in post-transplant maintenance for prevention of recurrence. However, the best method of chemotherapy is unknown, and whether ASCT should be included in first-line therapy remains unclear. Based on experience, allo-SCT is generally used in relapsed patients who failed in previous ASCT.

The French Société Française de Greffe de Moelle reported outcomes of RIC-allo-SCT in 73 patients with relapsed or refractory indolent lymphoma between 1998 and 2005^24^. The regimen generally used fludarabine with busulfan and ATG (*n*=43) and fludarabine with TBI (*n*=21). The median follow-up was 37 months (ranging from 16 to 77 months). In patients in CR, with partial response, and with chemoresistant disease, RR was 9.6%. The 3-year PFS rates were 66%, 52%, and 32%, respectively. The 3-year TRM rates were 32%, 28%, and 63%, respectively. Khouri *et al.*^23^ from MD Anderson studied 47 patients with FL. The median age was 53 years (ranging from 33 to 68 years). The patients underwent RIC regimen, which included fludarabine (30 mg/m^2^ daily for 3 days), cyclophosphamide (750 mg/m^2^ daily for 3 days), and rituximab (375 mg/m^2^ for 1 day plus 1,000 mg/m^2^ for 3 days on day -1, day +1, and day +8). Furthermore, 45 patients had sibling donors and 2 patients had unrelated donors. The estimated 5-year OS and PFS were 85% and 83%, respectively. With a median follow-up of 5 years, only 2 patients relapsed, and both responded to further treatment^23^. The outcomes illustrate that the RIC regimen can cure relapsed FL patients with HLA-identical sibling donor and are still sensitive to chemotherapy.

RIC-allo-SCT is an appropriate option for patients with advanced FL (early recurrence after intensive front-line or salvage therapy) who have a suitable HLA-matched sibling donor and minimal comorbidities. These promising results require further investigation in prospective multicenter trials.

### Aggressive B-cell lymphoma

For young patients with aggressive B-cell lymphoma, first-line treatment typically comprises 6 to 8 courses of R-CHOP or CHOP-like regimens given every 2 or 3 weeks. The majority of patients with diffused large B-cell lymphoma (DLBCL) and IPI (international prognostic index) 0-1 exhibit long-term survival after such therapy^25^, but the outcomes of patients with other histologic subtypes or higher IPI were less satisfactory. Patients resistant to treatment or those who relapsed after first-line therapy generally have poor prognosis, particularly if the relapse occurred early (<12 months) after primary therapy^26^. Patients who failed a variety of treatment options including auto-SCT are candidates for allo-SCT.

### Diffused large B-cell lymphoma

Although the treatment of DLBCL has significantly developed and the introduction of modern upfront immunochemotherapy protocols has greatly improved the outcomes of patients with DLBCL, many patients are still not cured using conventional therapy. ASCT remains the standard option for patients with relapsed disease. However, the outcomes of patients who relapse within 12 months after initial treatment with a rituximab-containing regimen are poor^27^. Patients who fail ASCT have limited options for effective treatment, so allo-SCT is a viable option.

Statistics of 68 patients from 23 transplant centers who underwent RIC-allo-SCT from October 1998 to January 2007 were analyzed in May 2008^28^. The median age of the cohort at transplant was 48 years (ranging from 17 to 66 years). Conditioning regimens were mostly based on fludarabine, which was combined with other chemotherapy drugs in 50 patients (74%) and TBI in 17 patients (25%). A portion of the patients (82%) had HLA-matched sibling donors, and 84% of the patients used stem cells from mobilized peripheral blood. The 2-year OS, PFS, and RR were 49%, 44%, and 41%, respectively. The cumulative incidences of 100-day and 1-year NRM were 14% and 23%, respectively. Based on univariate and multivariate analysis, the use of peripheral blood as stem cell source and CR status prior to transplantation were associated with a significant decrease in relapse incidence. Patients who developed cGVHD exhibited decreased incidence of relapse. Their findings demonstrated that RIC followed by allo-SCT can provide encouraging long-term survival in patients with relapsed or refractory DLCBL. EBMT reported an analysis of allo-SCT as salvage therapy for patients with DLBCL and relapse after ASCT. MAC-allo-SCT was used in 37 patients, and RIC-allo-SCT was used in 64 patients^29^. The 3-year NRM, RR, PFS, and OS were 28.2% (95% CI, 20% to 39%), 30.1% (95% CI, 22% to 41%), 41.7% (95% CI, 32% to 52%), and 53.8% (95% CI, 44% to 64%), respectively. NRM significantly increased in patients with an early relapse (<12 months) after ASCT and patients who were older than 45 years. RR was higher in refractory patients, and patients who relapsed after ASCT (<12 months) were associated with lower PFS. No difference was found between HLA-identical sibling donors and MUDs. Compared with MAC-allo-SCT, the outcomes of RIC-allo-SCT were associated with lower NRM and higher RR, with no differences in PFS and OS.

Based on these data, disease recurrence remains the primary cause of treatment failure. Efforts should be directed toward identifying the best RIC regimen to optimize the GVL effect. Protocols for GVHD prophylaxis should also be developed, and the optimal timing for transplantation should be determined.

### Mantle cell lymphoma (MCL)

MCL is a rare type of lymphoma. The pathophysiology and treatment of MCL has significantly developed over the last 2 decades, with a median OS of approximately 5 years to 6 years compared with only 2 years in the mid 1990s^30^. However, MCL remains an incurable disease characterized by repeated relapses. At present, the development of RIC-allo-SCT has resulted in the use of allo-SCT in a larger MCL population.

Cook *et al.*^31^ from the British Society of Blood and Marrow Transplantation registry analyzed a cohort of 70 MCL patients with a median age of 52.2 years (ranging from 34.7 to 68.8 years). The conditioning regimen consisted of fludarabine/melphalan (*n*=41), BEAM (BCNU, epotoside, cytarabine, melphalan) (*n*=22), and fludarabine/busulfan (*n*=7). Alemtuzumab was additionally administered to 52 patients. The median follow-up was approximately 37 months, and the 5-year OS, PFS, NRM, and cumulative risk of RRs were 37%, 14%, 21%, and 65%, respectively. The 3-year OS for patients in CR at transplantation were up to 60%. The RIC-allo-SCT option has to be discussed to all fit patients with an HLA-matched donor (related or not) experiencing relapse or refractory disease after appropriate first-line treatment with high-dose chemotherapy, and with a chemo-sensitive disease after salvage chemotherapy.

Pott *et al.*^32^ indicated that clinical relapse can be predicted by a positive minimal residual disease (MRD) level, which is measured by polymerase chain reaction (PCR) and/or flow cytometry techniques. The risk of relapse following RIC-allo-SCT raises the major question of immunomodulation following transplantation and MRD monitoring^33^. MCL patients should be encouraged to participate in prospective clinical trials to address the question of RIC-allo-SCT (**Table 2**).

**Table 2:** Results of RIC-allo-SCT in MCL

Author	Year	*n*	Median age (yr)	Disease status during transplantation	Conditioning regimen	PFS or EFS	OS	TRM
Robinson *et al.*^34^	2002	22	52 (44-57)	Chemosensitive 73%	Various	48.2% (1 y)	12.8% (2 y)	13.6% (100 d)
				Chemoresistant 14%		0% (2 y)		46% (1 y)
				unknown				82% (2 y)
Khouri *et al.*^35^	2003	18	56.5 (46-64)	CR 44%	Cisplatin/fludarabine/cytarabine (n=5);	33% (3 y)	45% (3 y)	0% (100 d)
				PR 44%	fludarabine/rituximab/cyclophosphamide (n=13)			
				SD 12%				
Morris *et al.*^36^	2004	10	48 (18-73)	CR 40%	Alemtuzumab/fludarabine/Melphalan	82% (3 y)	85.5% (3 y)	NRM 20% (3 y)
				PR 50%				
				Refractory 10%				
Armand *et al.*^37^	2008	15	51 (34-64)	CR 27%	Fludarabine/busulfan	50% (3 y)	60% (3 y)	NRM 37% (3 y)
				PR 49%				
				unknown				

CR, complete remission; PR, partial response; TBI, total body irradiation; PFS, progression-free survival; OS, overall survival; TRM, transplant-related mortality; NRM, non-relapse mortality.

### Aggressive T-cell lymphoma

Approximately 10% to 20% of lymphomas are of T- or natural killer-cell lineage. The outcomes for most mature T-cell lymphoma patients undergoing treatment are unsatisfactory, with high RRs^38^. In general, the prognosis is poor. Increasing data show the important function of RIC-allo-SCT in such patients.

Corradini *et al.*^39^ treated 17 patients with peripheral T-cell lymphomas (PTCL) using a reduced-intensity allogeneic procedure. They found high OS (81%) and PFS (64%) within three years. In this initial series, 15 out of 17 patients were chemosensitive before transplant. The two-year NRM rate was only 6%. In addition, the data for 35 patients was updated, with a median follow-up of 44 months. The PFS and OS of this series were 49% and 54%, respectively. Moreover, CR patients after transplant had 72% PFS compared with only 25% in patients with chemorefractory status at transplant. The PTCL report registered in the EBMT^40^ studied 91 patients who underwent RIC-allo-SCT with a prolonged follow-up of 50 months. The outcome was a 4-year PFS and OS of 39% and 43%, respectively. A plateau in the PFS and OS curve was observed after years from transplant. Similar satisfactory outcomes were reported by The Seattle Group^41^. Majority of patients with PTCL have lymphomas that cannot be further classified; thus, they are called PTCL not otherwise specified. Studies from several groups show high cure rates with allo-SCT in most patients using RIC^39^^,^^41^^,^^42^. High-risk PTCL patients who cannot benefit from frontline or salvage ASCT based on prognostic factors (i.e., a-IPI >1 and elevated b-2-microglobin)^43^ should be considered for RIC-allo-SCT. Based on recent studies, the poor prognosis for PTCL may change in a few years.

Adult T-cell leukemia-lymphoma (ATLL) results from chronic infection with the HTLV-1 virus, which can only be cured by allo-SCT when it’s aggressive. The Japanese Adult T-Cell Leukemia/Lymphoma Allo-SCT Study Group updated their initial results from 29 patients recruited to two consecutive prospective studies of RIC-allo-SCT^44^^-^^46^. The 5-year OS rate was 37%, with 10 long-term survivors (range of survival time, 54 to 100 months). Tanosaki *et al.*^45^ evaluated the feasibility of RIC-allo-SCT for 29 patients with ATLL. The regimen used fludarabine and busulfan, but the study particularly focused on the clinical impact of ATG. The 3-year OS and PFS were 36% and 31%, respectively. The HTLV-1 proviral load was undetectable by PCR in 62% of the patients. They concluded that RIC-allo-SCT using fludarabine and busulfan with or without low-dose ATG is feasible and safe even in elderly patients with ATLL. Hishizawa *et al.*^47^ showed that the use of HTLV-1-positive donors is associated with an increased rate of complications. Although these outcomes are encouraging, further prospective studies are needed to define the efficiency, indications, and impact of HTLV-1 proviral load on prognosis.

### Cutaneous T-cell lymphoma (CTCL)

CTCL are rare diseases that typically follow an indolent course. However, the prognosis is poor in advanced stages or transformation. Mycosis fungoides (MF) is the most common subtype of CTCL, but it only accounts for 0.5% of all NHLs (non hodgkin’s lymphomas)^48^. Se´zary syndrome (SS) is the erythrodermic or leukemic form of MF, in which malignant T cells circulate in the peripheral blood. This disease often has a poorer prognosis^49^. Several studies indicate that allo-SCT can be curative even in patients with refractory disease compared with ASCT^50^^,^^51^. Using meta-analysis, Wu *et al.*^52^ found that remissions are more frequent and more prolonged after allogeneic transplantation than after autologous transplantation.

A retrospective analysis by LWP of the EBMT reported the outcome of allogeneic transplantation for 36 patients with MF and 24 patients with SS between 1997 and 2007^50^, with median age of 46.5 years (ranging from 22 to 66 years). RIC regimen was administered to 44 patients, and 25 patients underwent T-cell depletion. The 1-year and 3-year OS rates were 66% and 54%, respectively, which were primarily influenced by donor type, disease phase, and type of conditioning. The RIC protocols decreased the risk of NRM to less than half the risk with MAC without increasing the risk of RR, resulting in higher OS rates of 73% and 63% at 1 and 3 years, respectively. Advanced-phase disease increased RR and reduced PFS and OS. Patients who received allo-SCT from a matched related donor had higher PFS and OS than their unrelated donor counterparts. Although the recipients of RIC protocols were older than those of MAC-allo-SCT, RIC-allo-SCT had higher OS than MAC-allo-SCT when the analysis was restricted to patients younger than  50 years. Across all the outcomes, the widest impact factor of the disease was disease status. Their data suggest that the use of allo-SCT in CTCL is promising. Herbert *et al.*^53^ studied the GVL effect on refractory CTCL after reduced-intensity HLA-matched sibling allo-SCT. They posited that although allo-SCT is a valid therapeutic alternative for high-risk patients with advanced-stage MF/SS, this method should be further improved. When considering allo-SCT for patients with advanced disease (stage III/IV or transformed disease), this treatment should be used relatively early in the disease course to increase long-term PFS and OS. Furthermore, although RIC-allo-SCT potentially reduces treatment toxicity, very few patients still die from the complications of GVHD. Therefore, this procedure should be further improved to control the severity of GVHD, particularly in patients who are at high risk of infection. The degree of the disease in these patients may have been highly extensive for the GVL effect to control the disease. Duvic *et al.*^51^ studied the safety and efficacy of total skin electron beam (TSEB) with RIC-allo-SCT in patients with CTCL. TSEB followed by RIC-allo-SCT was applied to 19 patients with advanced CTCL (median age, 50 years) between July 2001 and July 2008. Fludarabine (125 mg/m^2^) and melphalan (140 mg/m^2^) plus thymoglobulin (for mismatched donors) were used to condition 16 patients. Tacrolimus/mini methotrexate was used for GVHD prophylaxis. In August 2009, 6 (31%) of the 19 patients died and 4 of the 6 patients were in CR. The 2-year NRM, OS, and PFS were 88% (95% CI, 74% to 100%), 79% (95% CI, 65% to 100%), and 53% (95% CI, 31% to 92%), respectively. Therefore, the combination of TSEB with RIC-allo-SCT is an effective therapy for patients with refractory CTCL. This procedure should be further studied in high-risk patients with advanced disease, poor survival, and matched donors.

MF occurs at an average age of 60 years, and the use of RIC regimen renders allo-SCT feasible for elderly patients with MF/SS and patients with reduced organ capacity after multiple lines of therapy, including patients in their seventies. Moreover, RIC-allo-SCT has better outcomes even in patients younger than 50 years^50^ and may be an option for the majority of patients.

## Conclusion and perspectives

In summary, RIC-allo-SCT is a feasible option that has the potential to induce long-term remissions for various subtypes of malignant lymphoma. RIC-allo-SCT can accelerate bone marrow suppression after transplantation, reduce the infections caused by agranulocytosis, and enhance the GVL effect. Thus, this method has many application prospects. Despite the great progress in theoretical and clinical applications and generally satisfactory outcomes of this method, further clinical research should be conducted. Retrospective studies suggest that RIC regimens render allo-SCT appropriate for patients because MAC-allo-SCT is associated with high risks of NRM. The control of incidence is also unsatisfactory. Therefore, new strategies are needed to settle this problem. Further studies are needed to ascertain the optimal timing, the best RIC regimen for allo-SCT, whether RIC-allo-SCT can completely replace MAC-allo-SCT, and so on. Studies that aim to determine which patients might benefit the most from RIC-allo-SCT and maximize the GVL effect while minimizing the incidence and the severity of acute GVHD are underway.

## References

[1] Chopra R Goldstone AH Pearce R Philip T Petersen F Appelbaum F Autologous versus allogeneic bone marrow transplantation for non-Hodgkin’s lymphoma: a case-controlled analysis of the European Bone Marrow Transplant Group Registry data. J Clin Oncol 1992;10:1690-1695 1403052 10.1200/JCO.1992.10.11.1690

[2] Verdonck LF Dekker AW Lokhorst HM Petersen EJ Nieuwenhuis HK Allogeneic versus autologous bone marrow transplantation for refractory and recurrent low-grade non-Hodgkin’s lymphoma. Blood 1997;90:4201-4205 9354692

[3] van Besien K Loberiza FR Jr Bajorunaite R Armitage JO Bashey A Burns LJ Comparison of autologous and allogeneic hematopoietic stem cell transplantation for follicular lymphoma. Blood 2003;102:3521-3529 12893748 10.1182/blood-2003-04-1205

[4] Sureda A Robinson S Canals C Carella AM Boogaerts MA Caballero D Reduced-intensity conditioning compared with conventional allogeneic stem-cell transplantation in relapsed or refractory Hodgkin’s lymphoma: an analysis from the Lymphoma Working Party of the European Group for Blood and Marrow Transplantation. J Clin Oncol 2008;26:455-462 18086796 10.1200/JCO.2007.13.2415

[5] Schmitz N Dreger P Glass B Sureda A. Allogeneic transplantation in lymphoma: current status. Haematologica 2007;92:1533-1548 18024402 10.3324/haematol.11185

[6] Linch DC Winfield D Goldstone AH Moir D Hancock B McMillan A Dose intensification with autologous bone-marrow transplantation in relapsed and resistant Hodgkin’s disease: results of a BNLI randomised trial. Lancet 1993;341:1051-1054 8096958 10.1016/0140-6736(93)92411-l

[7] Milpied N Fielding AK Pearce RM Ernst P Goldstone AH Allogeneic bone marrow transplant is not better than autologous transplant for patients with relapsed Hodgkin’s disease. European Group for Blood and Bone Marrow Transplantation. J Clin Oncol 1996;14:1291-1296 8648386 10.1200/JCO.1996.14.4.1291

[8] Robinson SP Sureda A Canals C Russell N Caballero D Bacigalupo A Reduced intensity conditioning allogeneic stem cell transplantation for Hodgkin’s lymphoma: identification of prognostic factors predicting outcome. Haematologica 2009;94:230-238 19066328 10.3324/haematol.13441 PMC2635413

[9] Sureda A Canals C Arranz R Caballero D Ribera JM Brune M Allogeneic stem cell transplantation after reduced intensityconditioning in patients with relapsed or refractory Hodgkin’slymphoma. Results of the HDR-ALLO study-a prospectiveclinical trial by the Grupo Espanol de Linfomas/ Trasplante deMedula Osea (GEL/TAMO) and the Lymphoma Working Party ofthe European Group for Blood and Marrow Transplantation. Haematologica 2012;97:310-7 21993674 10.3324/haematol.2011.045757 PMC3269494

[10] Montserrat E Moreno C Esteve J Urbano-Ispizua A Gine E Bosch F. How I treat refractory CLL. Blood 2006;107:1276-1283 16204307 10.1182/blood-2005-02-0819

[11] Hallek M Fingerle-Rowson G Fink A. Immunochemotherapy with fludarabine, cyclophosphamide, and Rituximab (FCR) versus fludarabine and cyclophosphamide (FC) improves response rates and progression-free survival (PFS) of previously untreated patients (pts) with advanced chronic lymphocytic leukemia abstract. Blood 2008;112:125

[12] Dreger P Corradini P Kimby E Michallet M Milligan D Schetelig J Indications for allogeneic stem cell transplantation in chronic lymphocytic leukemia: the EBMT transplant consensus. Leukemia 2007;21:12-17 17109028 10.1038/sj.leu.2404441

[13] Dreger P. Allotransplantation for chronic lymphocytic leukemia. Hematology Am Soc Hematol Educ Program 2009:602-609 20008245 10.1182/asheducation-2009.1.602

[14] Schetelig J Thiede C Bornhauser M Schwerdtfeger R Kiehl M Beyer J Evidence of a graft-versus-leukemia effect in chronic lymphocytic leukemia after reduced-intensity conditioning and allogeneic stem-cell transplantation: the Cooperative German Transplant Study Group. J Clin Oncol 2003;21:2747-2753 12860954 10.1200/JCO.2003.12.011

[15] Brown JR Kim HT Li S Stephans K Fisher DC Cutler C Predictors of improved progression-free survival after nonmyeloablative allogeneic stem cell transplantation for advanced chronic lymphocytic leukemia. Biol Blood Marrow Transplant 2006;12:1056-1064 17084369 10.1016/j.bbmt.2006.06.004

[16] Delgado J Thomson K Russell N Ewing J Stewart W Cook G Results of alemtuzumab-based reduced-intensity allogeneic transplantation for chronic lymphocytic leukemia: a British Society of Blood and Marrow Transplantation Study. Blood 2006; 107: 1724-1730 16239425 10.1182/blood-2005-08-3372

[17] Khouri IF Saliba RM Admirand J O’Brien S Lee MS Korbling M Graft-versus-leukaemia effect after non-myeloablative haematopoietic transplantation can overcome the unfavourable expression of ZAP-70 in refractory chronic lymphocytic leukaemia. Br J Haematol 2007;137:355-363 17456058 10.1111/j.1365-2141.2007.06591.x

[18] Sorror ML Storer BE Sandmaier BM Maris M Shizuru J Maziarz R Five-year follow-up of patients with advanced chronic lymphocytic leukemia treated with allogeneic hematopoietic cell transplantation after nonmyeloablative conditioning. J Clin Oncol 2008;26:4912-4920 18794548 10.1200/JCO.2007.15.4757 PMC2652085

[19] Dreger P Dohner H Ritgen M Bottcher S Busch R Dietrich S Allogeneic stem cell transplantation provides durable disease control in poor-risk chronic lymphocytic leukemia: long-term clinical and MRD results of the German CLL Study Group CLL3X trial. Blood 2010;116:2438-2447 20595516 10.1182/blood-2010-03-275420

[20] Schetelig J van Biezen A Brand R Caballero D Martino R Itala M Allogeneic hematopoietic stem-cell transplantation for chronic lymphocytic leukemia with 17p deletion: a retrospective European Group for Blood and Marrow Transplantation analysis. J Clin Oncol 2008;26:5094-5100 18711173 10.1200/JCO.2008.16.2982

[21] Delgado J Pillai S Benjamin R Caballero D Martino R Nathwani A The effect of in vivo T cell depletion with alemtuzumab on reduced-intensity allogeneic hematopoietic cell transplantation for chronic lymphocytic leukemia. Biol Blood Marrow Transplant 2008;14:1288-1297 18940684 10.1016/j.bbmt.2008.09.001

[22] Herold M Haas A Srock S Neser S Al-Ali KH Neubauer A Rituximab added to first-line mitoxantrone, chlorambucil, and prednisolone chemotherapy followed by interferon maintenance prolongs survival in patients with advanced follicular lymphoma: an East German Study Group Hematology and Oncology Study. J Clin Oncol 2007;25:1986-1992 17420513 10.1200/JCO.2006.06.4618

[23] Khouri IF McLaughlin P Saliba RM Hosing C Korbling M Lee MS Eight-year experience with allogeneic stem cell transplantation for relapsed follicular lymphoma after nonmyeloablative conditioning with fludarabine, cyclophosphamide, and rituximab. Blood 2008;111:5530-5536 18411419 10.1182/blood-2008-01-136242 PMC4624452

[24] Vigouroux S Michallet M Porcher R Attal M Ades L Bernard M Long-term outcomes after reduced-intensity conditioning allogeneic stem cell transplantation for low-grade lymphoma: a survey by the French Society of Bone Marrow Graft Transplantation and Cellular Therapy (SFGM-TC). Haematologica 2007;92:627-634 17488686 10.3324/haematol.10924

[25] Pfreundschuh M Trumper L Osterborg A Pettengell R Trneny M Imrie K CHOP-like chemotherapy plus rituximab versus CHOP-like chemotherapy alone in young patients with good-prognosis diffuse large-B-cell lymphoma: a randomised controlled trial by the MabThera International Trial (MInT) Group. Lancet Oncol 2006;7:379-391 16648042 10.1016/S1470-2045(06)70664-7

[26] Blay J Gomez F Sebban C Bachelot T Biron P Guglielmi C The International Prognostic Index correlates to survival in patients with aggressive lymphoma in relapse: analysis of the PARMA trial. Parma Group. Blood 1998;92:3562-3568 9808548

[27] Gisselbrecht C Glass B Mounier N Singh Gill D Linch DC Trneny M Salvage regimens with autologous transplantation for relapsed large B-cell lymphoma in the rituximab era. J Clin Oncol 2010;28:4184-4190 20660832 10.1200/JCO.2010.28.1618 PMC3664033

[28] Sirvent A Dhedin N Michallet M Mounier N Faucher C Yakoub-Agha I Low nonrelapse mortality and prolonged long-term survival after reduced-intensity allogeneic stem cell transplantation for relapsed or refractory diffuse large B cell lymphoma: report of the Societe Francaise de Greffe de Moelle et de Therapie Cellulaire. Biol Blood Marrow Transplant 2010;16:78-85 19744569 10.1016/j.bbmt.2009.09.002

[29] van Kampen RJ Canals C Schouten HC Nagler A Thomson KJ Vernant JP Allogeneic stem-cell transplantation as salvage therapy for patients with diffuse large B-cell non-Hodgkin’s lymphoma relapsing after an autologous stem-cell transplantation: an analysis of the European Group for Blood and Marrow Transplantation Registry. J Clin Oncol 2011;29:1342-1348 21321299 10.1200/JCO.2010.30.2596

[30] Herrmann A Hoster E Zwingers T Brittinger G Engelhard M Meusers P Improvement of overall survival in advanced stage mantle cell lymphoma. J Clin Oncol 2009;27:511-518 19075279 10.1200/JCO.2008.16.8435

[31] Cook G Smith GM Kirkland K Lee J Pearce R Thomson K Outcome following Reduced-Intensity Allogeneic Stem Cell Transplantation (RIC AlloSCT) for relapsed and refractory mantle cell lymphoma (MCL): a study of the British Society for Blood and Marrow Transplantation. Biol Blood Marrow Transplant 2010;16:1419-1427 20399879 10.1016/j.bbmt.2010.04.006

[32] Pott C Schrader C Gesk S Harder L Tiemann M Raff T Quantitative assessment of molecular remission after high-dose therapy with autologous stem cell transplantation predicts long-term remission in mantle cell lymphoma. Blood 2006;107:2271-2278 16332971 10.1182/blood-2005-07-2845

[33] Le Gouill S Mohty M Guillaume T Gastinne T Moreau P. Allogeneic stem cell transplantation in mantle cell lymphoma: where are we now and which way should we go? Semin Hematol 2011;48:227-239 21782065 10.1053/j.seminhematol.2011.03.009

[34] Robinson SP Goldstone AH Mackinnon S Carella A Russell N de Elvira CR Chemoresistant or aggressive lymphoma predicts for a poor outcome following reduced-intensity allogeneic progenitor cell transplantation: an analysis from the Lymphoma Working Party of the European Group for Blood and Bone Marrow Transplantation. Blood 2002;100:4310-4316 12393626 10.1182/blood-2001-11-0107

[35] Khouri IF Lee MS Saliba RM Jun G Fayad L Younes A Nonablative allogeneic stem-cell transplantation for advanced/recurrent mantle-cell lymphoma. J Clin Oncol 2003;21:4407-4412 14645431 10.1200/JCO.2003.05.501

[36] Morris E Thomson K Craddock C Mahendra P Milligan D Cook G Outcomes after alemtuzumab-containing reduced-intensity allogeneic transplantation regimen for relapsed and refractory non-Hodgkin lymphoma. Blood 2004;104:3865-3871 15304395 10.1182/blood-2004-03-1105

[37] Armand P Kim HT Ho VT Cutler CS Koreth J Antin JH Allogeneic transplantation with reduced-intensity conditioning for Hodgkin and non-Hodgkin lymphoma: importance of histology for outcome. Biol Blood Marrow Transplant 2008;14:418-425 18342784 10.1016/j.bbmt.2008.01.008 PMC2364453

[38] Armitage JO Hsi ED Foss FM Clinical roundtable monograph. T-cell lymphoma: therapeutic overview and disease state awareness. Clin Adv Hematol Oncol 2010;8:1-15 21491667

[39] Corradini P Dodero A Zallio F Caracciolo D Casini M Bregni M Graft-versus-lymphoma effect in relapsed peripheral T-cell non-Hodgkin’s lymphomas after reduced-intensity conditioning followed by allogeneic transplantation of hematopoietic cells. J Clin Oncol 2004;22:2172-2176 15169805 10.1200/JCO.2004.12.050

[40] Gutiérrez A Caballero MD Perez-Manga G Rodriguez J Hematopoietic SCT for peripheral T-cell lymphoma. Bone Marrow Transplant 2008;42:773-781 18936735 10.1038/bmt.2008.332

[41] Shustov AR Gooley TA Sandmaier BM Shizuru J Sorror ML Sahebi F Allogeneic haematopoietic cell transplantation after nonmyeloablative conditioning in patients with T-cell and natural killer-cell lymphomas. Br J Haematol 2010;150:170-178 20507311 10.1111/j.1365-2141.2010.08210.x PMC2995443

[42] Zain J Palmer JM Delioukina M Thomas S Tsai NC Nademanee A Allogeneic hematopoietic cell transplant for peripheral T-cell non-Hodgkin lymphoma results in long-term disease control. Leuk Lymphoma 2011;52:1463-1473 21699453 10.3109/10428194.2011.574754 PMC3441144

[43] Rodríguez J Conde E Gutierrez A Lahuerta JJ Arranz R Sureda A The adjusted International Prognostic Index and beta-2-microglobulin predict the outcome after autologous stem cell transplantation in relapsing/refractory peripheral T-cell lymphoma. Haematologica 2007;92:1067-1074 17640855 10.3324/haematol.11173

[44] Choi I Tanosaki R Uike N Utsunomiya A Tomonaga M Harada M Long-term outcomes after hematopoietic SCT for adult T-cell leukemia/lymphoma: results of prospective trials. Bone Marrow Transplant 2011;46:116-118 20400987 10.1038/bmt.2010.92

[45] Tanosaki R Uike N Utsunomiya A Saburi Y Masuda M Tomonaga M Allogeneic hematopoietic stem cell transplantation using reduced-intensity conditioning for adult T cell leukemia/lymphoma: impact of antithymocyte globulin on clinical outcome. Biol Blood Marrow Transplant 2008;14:702-708 18489996 10.1016/j.bbmt.2008.03.010

[46] Okamura J Utsunomiya A Tanosaki R Uike N Sonoda S Kannagi M Allogeneic stem-cell transplantation with reduced conditioning intensity as a novel immunotherapy and antiviral therapy for adult T-cell leukemia/lymphoma. Blood 2005;105:4143-4145 15665110 10.1182/blood-2004-11-4193

[47] Hishizawa M Kanda J Utsunomiya A Taniguchi S Eto T Moriuchi Y Transplantation of allogeneic hematopoietic stem cells for adult T-cell leukemia: a nationwide retrospective study. Blood 2010;116:1369-1376 20479287 10.1182/blood-2009-10-247510

[48] Kim YH Hoppe RT Mycosis fungoides and the Sezary syndrome. Semin Oncol 1999;26:276-289 10375085

[49] Willemze R Jaffe ES Burg G Cerroni L Berti E Swerdlow SH WHO-EORTC classification for cutaneous lymphomas. Blood 2005;105:3768-3785 15692063 10.1182/blood-2004-09-3502

[50] Duarte RF Canals C Onida F Gabriel IH Arranz R Arcese W Allogeneic hematopoietic cell transplantation for patients with mycosis fungoides and Sezary syndrome: a retrospective analysis of the Lymphoma Working Party of the European Group for Blood and Marrow Transplantation. J Clin Oncol 2010;28:4492-4499 20697072 10.1200/JCO.2010.29.3241

[51] Duvic M Donato M Dabaja B Richmond H Singh L Wei W Total skin electron beam and non-myeloablative allogeneic hematopoietic stem-cell transplantation in advanced mycosis fungoides and Sezary syndrome. J Clin Oncol 2010;28:2365-2372 20351328 10.1200/JCO.2009.25.8301

[52] Wu PA Kim YH Lavori PW Hoppe RT Stockerl-Goldstein KE A meta-analysis of patients receiving allogeneic or autologous hematopoietic stem cell transplant in mycosis fungoides and Sezary syndrome. Biol Blood Marrow Transplant 2009;15:982-990 19589488 10.1016/j.bbmt.2009.04.017 PMC6402786

[53] Herbert KE Spencer A Grigg A Ryan G McCormack C Prince HM Graft-versus-lymphoma effect in refractory cutaneous T-cell lymphoma after reduced-intensity HLA-matched sibling allogeneic stem cell transplantation. Bone Marrow Transplant 2004;34:521-525 15286686 10.1038/sj.bmt.1704641

